# *In vivo* photoacoustic tomography of porcine abdominal organs using Fabry–Pérot sensing integrated platform

**DOI:** 10.1186/s41747-025-00601-1

**Published:** 2025-07-09

**Authors:** Damien Gasteau, Alexis Vrignaud, Arnaud Biallais, Fabrice Richard, Gilles Blancho, Julien Branchereau, Benoît Mesnard

**Affiliations:** 1 DeepColor Imaging SAS, Nantes, France; 2 Nantes Université, CHU Nantes1, INSERM, Centre for Research in Transplantation and Translational Immunology, Nantes, France; 3 Department of Urology and Transplantation Surgery, Nantes University Hospital, Nantes, France

**Keywords:** Anatomy, Animal, Disease model, Equipment, Photoacoustic techniques

## Abstract

**Objective:**

To evaluate *in vivo* a fully integrated photoacoustic tomography imaging system based on Fabry–Pérot ultrasound sensing method applied on porcine abdominal organs. This approach could be used by surgeons during intraoperative clinical procedures.

**Methods:**

The photoacoustic imaging system was fully integrated into a single structure, and the detection technology was based on a Fabry–Pérot interferometer. The detection probe connected to the imaging system was applied directly to the organs of a male “large white” *Sus scrofa* pig weighing 80 kg, either manually or using a stand, with or without a gel interface. All experiments were performed in compliance with EU Directive 2010/63/EU on animal experimentation (APAFiS #31507).

**Results:**

All intraperitoneal and retroperitoneal organs were evaluated using photoacoustic imaging. The evaluation of both hollow and solid organs was successfully conducted with consistent three-dimensional image quality. We demonstrate the system’s ability to image blood vessels with diameters ranging from several millimeters down to less than 100 µm. Macroscopic evaluation of the organs using photoacoustic tomography imaging did not reveal any damage or burns caused by the excitation laser.

**Conclusion:**

To our knowledge, this is the first reported imaging session of abdominal organs in an *in vivo* porcine model, performed using a photoacoustic tomography system with Fabry–Pérot interferometer detection. We present a high-resolution photoacoustic tomography system that is closer to routine clinical translation, thanks to a fully integrated system.

**Relevance statement:**

Photoacoustic evaluation of organs using a fully integrated system could become a valuable tool for surgical teams for intraprocedural assessment of vascularization.

**Key Points:**

- Photoacoustic imaging visualizes blood vessels without contrast agents or ionizing radiation.
- Photoacoustic imaging systems detect blood vessels ranging from millimeters to 100 µm.
- Fully integrated photoacoustic imaging systems are autonomously operable by surgical teams.

**Graphical Abstract:**

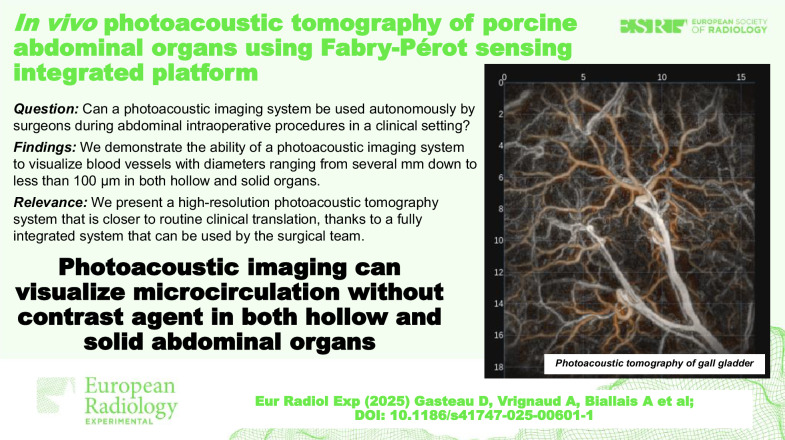

## Background

Photoacoustic imaging is an innovative modality that is increasingly gaining interest in biomedical research. It combines optical and ultrasound technologies to provide high-resolution vascular imaging and has potential for various clinical applications. Among emerging technologies designed to assist surgeons during intervention, photoacoustic tomography imaging (PATI) offers a unique perspective due to its ability to visualize blood vessels without requiring an exogenous contrast agent or exposing patients to ionizing radiation. PATI enables the visualization of optical absorber distributions at greater depths than purely optical methods, such as fluorescence, by utilizing weakly scattering ultrasound waves. While still a rapidly evolving imaging modality [1–3], PATI is already able to not only be a simple guidance tool by revealing the location of buried vessels but also can provide important information such as hemodynamics [4] or organ health [5].

This technique relies on short (nanosecond) light pulses that induce rapid thermal expansion through the optical absorption of local chromophores. The transient rise and decay of localized stresses generate ultrasound waves within the tissue. These acoustic signals can then be captured, allowing the reconstruction of an image of the acoustic sources. The development of an optimized setup that is clinically viable, particularly for use during surgery, remains a challenging task. Ultrasound detection and illumination configurations are critical features distinguishing PATI systems. Among detection technologies, Fabry–Pérot interferometer (FPI) ultrasound detection emerges as a well-suited solution for PATI. Its large broadband response covering with a single sensor the typical frequency range of ultrasound imaging (2–15 MHz), high density and number of ultrasound detection areas (typically 100 sensors/mm², totaling 40,000 sensors), and sensitivity enable higher photoacoustic image quality in comparison with piezoelectric based detection methods [6–9] by revealing both large and small features with high contrast. Furthermore, its transparency in the near-infrared wavelength range eliminates illumination conflicts, allowing the sensor to be placed directly against the tissue and obviating the need for a coupling liquid recess [10, 11]. However, compared to other common detection hardware, such as piezoelectric elements, challenges persist in terms of fabrication, integration, interrogation, and speed performance.

The limited number of PATI studies conducted on human organs prevents a comprehensive understanding of the applicability of this technology. The porcine model provides a suitable preclinical research platform for imaging studies of human intra-abdominal organs due to its anatomical and physiological similarities. Previous studies have shown that the porcine model has been successfully used in the validation and optimization stages of various imaging methods. However, experimental research on PATI of intra-abdominal organs is still limited. The objectives of our study were: to evaluate a new fully integrated photoacoustic imaging system based on FPI ultrasound sensing, designed for autonomous clinical use by surgeons during intraoperative procedures; to examine how different intra-abdominal organs can be imaged with PATI; to identify the technical challenges associated with the method; and to assess the limitations encountered during the imaging process. Furthermore, we aim to contribute to the potential clinical application of PATI for intraabdominal organs by analyzing the anatomical accuracy of the obtained images.

## Methods

We conducted a preclinical exploratory study using a porcine model. As part of an experimental renal transplantation study, a PATI session was performed on the intraabdominal organs prior to the organ procurement. One by one, PATI was performed under aseptic conditions identical to those of an operating room.

### Porcine model and animal ethics

Given the close anatomical and physiological resemblance between the abdominal organs of pigs and humans [12], we selected a porcine model for our study. A male “large white” *Sus scrofa* pig weighing 80 kg was used to study vascular anatomy, approximating that of an adult human subject. A premedication was performed with Zolazepam/Tiletamine 15 mg/kg intramuscularly. Then the pig was subsequently intubated and ventilated with a mixture of sevoflurane (2%), nitrous oxide (49%), and oxygen (49%) to induce general anesthesia. The gas flow rate was 2 L/min, and the respiratory rate was 20 breaths/min. Perioperative analgesia was performed by intravenous injection of nalbuphine and paracetamol at 25 mg/kg. Mean arterial pressure, pulse rate, heart rate, oxygen saturation, and temperature were monitored during the surgical procedure. A laparotomy was performed through an xypho-pubic incision to expose the abdominal and the retroperitoneal organs. PATI was then performed successively on the abdominal viscera. Ultrasound gel was interposed only in the absence of full contact with the FPI. When the abdominal movements were too important for the acquisition, the ventilation of the animal was paused for a few seconds while ensuring that the oxygen saturation was maintained above 95%. After the acquisition of all data, the initial protocol was resumed with a multi-organ procurement and terminal euthanasia.

The experiment was conducted in association with and indexed to a previous research protocol (multiorgan procurement under general anesthesia with a no-wake-up procedure). The study protocol was approved by the French Research Ministry (APAFiS #31507). All experiments adhered to the ARRIVE guidelines [13] and were performed in compliance with EU Directive 2010/63/EU on animal experimentation. Internal organ imaging exposition thresholds are not well known and could potentially be increased for some organs [14, 15]. To prevent organ burns associated with laser emission, this work was achieved following the exposure limitations level of skin as a baseline (IEC 60825).

### Imaging platform

**Fig. 1:**
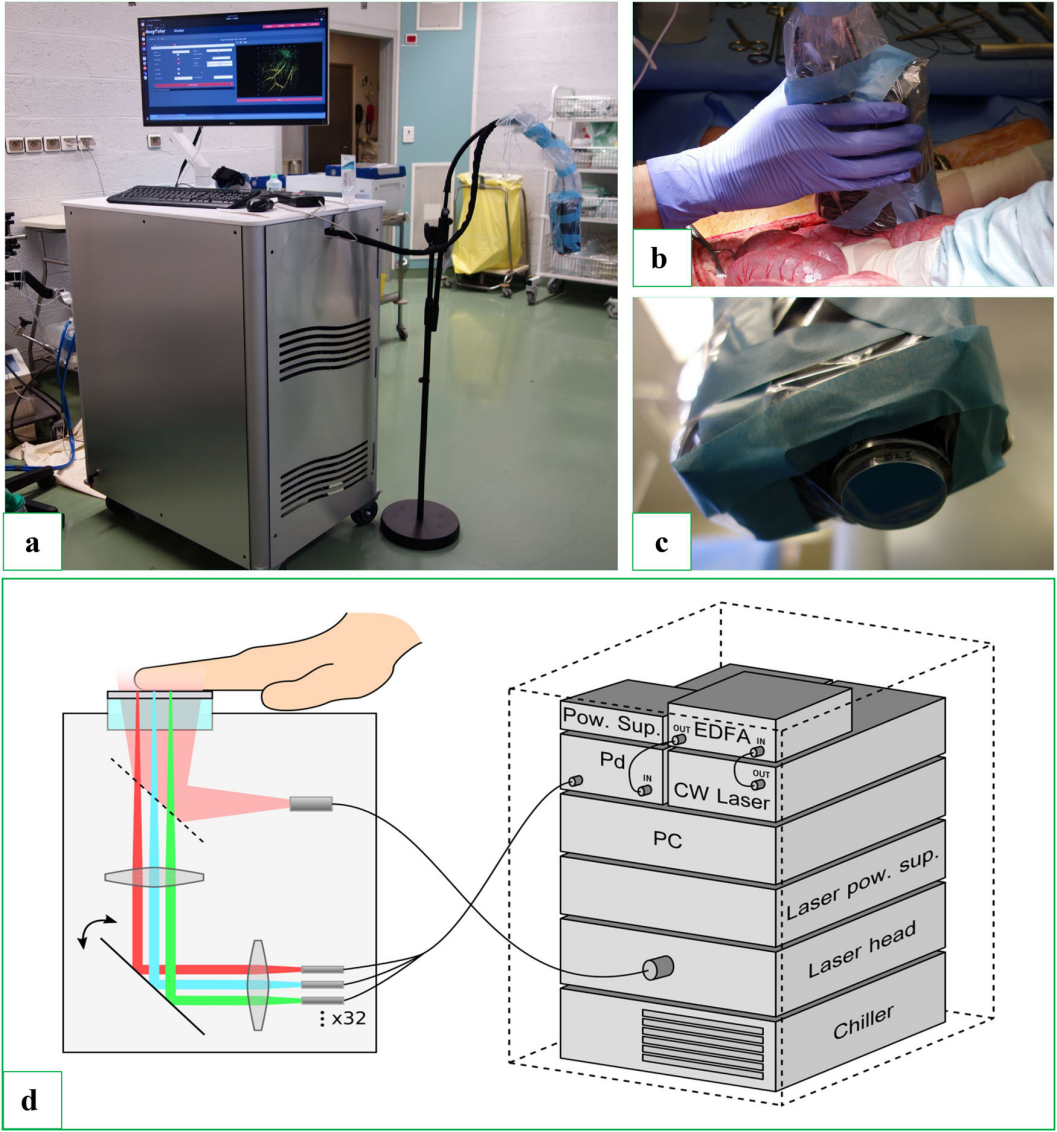
**a** Photoacoustic tomography prototype. The imaging probe can be seen wrapped in a plastic bag on a stand. **b** The probe when held by the surgeon performing the images. The organ imaged was the colon wall. **c** Close-up of the probe and the FPI sensor. **d** Schematic representation of the photoacoustic tomography system, detailing the components included in the system. CW Laser, Continuous wave laser; EDFA, Erbium-doped fiber amplifier; FPI, Fabry–Pérot interferometer; PC, Integrated computer; Pd, Photodiode chain; Pow. Sup., Power supply

The imaging system used in this study was a prototype provided by DeepColor SAS. As illustrated in Fig. 1, the system was fully integrated into a single structure (approximately 80 × 60 × 110 cm, excluding the monitor) and featured an OPO laser (Photosonus X-U19) for photoacoustic generation, with a pulse duration of 4.5 ns and a repetition rate of 100 Hz, and its corresponding chiller. The excitation laser was set to emit at 800 nm and 18 mJ per pulse with a Gaussian intensity profile filling the sensor's 30-mm diameter. A PC for hardware control and image reconstruction was placed under the interrogation rack described below. A big size gain was achieved with the interrogation unit, including the continuous laser source, amplifier, optical circulators, photodiodes, and custom amplification stages. The prototype incorporated technical advancements as described in Ansari et al [16], Huynh et al [17], and Zhang et al [11]. Compared to the latest work from Huynh et al [18], the size was almost halved with an overall more optimized design. It included a compact handheld probe (4.5 × 10.2 × 15.5 cm^3^), shown in Fig. 1b wrapped in a protective sleeve, and connected to the main structure via flexible cabling, significantly enhancing ease of use compared to earlier research-oriented setups. Its integration into a handheld design simplified manipulation and image acquisition in clinical conditions. To further improve image quality, motion blur could be minimized by mounting the probe on a stand with an adapter piece.

The probe incorporated the FPI ultrasound sensor, the photoacoustic illumination bundle output, and optical components for focusing and steering detection beams onto the FPI surface and returning them to their respective fibers. A visible-range camera was also included for imaging area visualization, though it was not utilized in the current study. The system employs a tunable semiconductor laser (TSL 550, Santec) to generate the detection beam, which is split into 32 parallel beams focused on the FPI surface within the probe. Reflected light is redirected to individual InGaS photodiodes, and acoustic traces were acquired at a 60 MHz sampling rate without averaging. The 32 beams were arranged linearly and steered incrementally to perform a stepped scan of the FPI sensor, with each step synchronized with a photoacoustic excitation laser pulse. At a pulse repetition frequency (PRF) of 100 Hz, the system achieved a scanning rate of 3,200 A-lines per second. This configuration eliminates the need for probe translation during data acquisition. Single images were acquired in approximately 10 s, using 100 µm step sizes over a 19 × 16 mm^2^ sensor area. The effective field of view could exceed the sensor area due to the FPI sensor’s detection angle.

The sensitivity of detection points varied across the FPI sensor due to manufacturing and optical constraints. While software correction partially mitigated these inhomogeneities, artifacts may still appear in some images, particularly in the spleen image provided in the Supplementary Materials (Appendix S1).

### Volume reconstruction

The acquisition software enabled visualization within seconds, allowing the final three-dimensional (3D) image to be presented to the surgical team. The image quality obtained was sufficient to identify vascularization and provide a vascular circulation map. However, it may present artifacts that require offline rework using the following process. Acquisition, processing, reconstruction, visualization, and database management were performed with a beta version (v 0.8.10) of DeepColor’s acquisition software. It was developed in-house, leveraging open-source languages, such as Nim 1.6, Python 3.8, and Julia 1.7. Two-dimensional visualizations were performed with the Julia library Makie, and 3D visualizations were performed with the PyVista library. Prior to reconstruction, acoustic traces were filtered using a high-pass filter and wavelet filtering to partially denoise signals. The initial pressure field was reconstructed using the “fast Fourier transform” approach [19] immediately after acquisition during the imaging session for fast results. Subsequently, an iterative time reversal algorithm including acoustic attenuation [20] was used to obtain the final volumes presented in the following manuscript. Optimal speed of sound was chosen using an autofocus approach, maximizing the global gradient of the image [21]. Postprocessing inhomogeneous fluence due to the Gaussian illumination profile of the excitation laser beam was compensated. Depth light intensity decay was corrected using an exponential approximation in the form exp(-μeff), where μeff was manually adjusted in the software interface. Further image quality enhancing techniques were applied, such as sigmoid normalization [22] and logarithmic compression [23].

To visualize the 3D reconstructed volume, two approaches were used in this paper: maximum intensity projection (MIP), and 3D volume rendering. MIPs deliver an overall appreciation of the 3D volume by projecting in the visualization plane the voxel with the strongest intensity along the normal axis. It was completed with depth color encoding to distinguish the depth location of the respective voxels. Secondly, 3D volume rendering was used as it allowed for freely manipulating the volume orientation. The corresponding transparency function was correlated to the voxel intensity to put emphasis on strong features and eliminate low-intensity voxels. Generally, the first layers, depth-wise, of the rendering were cropped as they contain only artifacts and or bubbles.

## Results

All intraperitoneal and retroperitoneal organs were evaluated using PATI. All acquisitions were performed without the use of ultrasound gel, relying instead on the natural interface provided by peritoneal fluid, except for the ureter. Ventilation was maintained during the imaging session, except during the acquisition of images of the liver and stomach, which lasted only a few seconds each. These two organs are particularly subject to large breathing-induced movement due to their close vicinity to the lungs. All the PATI images presented are derived from a single acquisition. No images were re-acquired to ensure that the data reflect real-life usage of this PATI system. Following the imaging session, macroscopic evaluation of the organs using PATI did not reveal any damage or burns caused by the excitation laser.

### Hollow organs

**Fig. 2:**
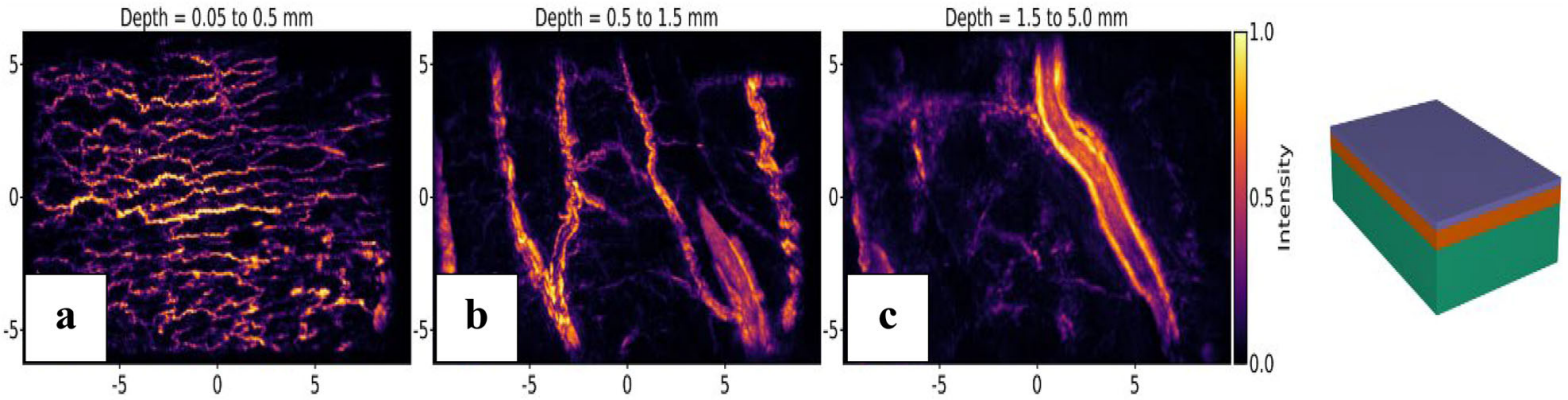
MIPs of the reconstructed volume of stomach tissue. **a** Reconstruction between 0.05 mm and 0.5 mm of depth. **b** Reconstruction between 0.5 mm and 1.5 mm of depth. **c** Reconstruction between 1.5 mm and 5 mm of depth. The superficial layer formed a web of small vessels mapping the tissue, while the deeper layers consisted of larger, branching vessels

Hollow organs, such as the stomach, small intestine, colon, rectum, gall bladder, bladder, or ureter, typically exhibited a maximal wall thickness of only a few millimeters. These walls contained several layers of blood vessels, many of which were not visible to the naked eye. PATI imaging of the stomach tissue, shown in Fig. 2, revealed three distinct layers of overlapping vessel networks. Each layer exhibited a unique vascular texture: the superficial layer formed a web of small vessels mapping the tissue, while the deeper layers consisted of larger, branching vessels.

**Fig. 3:**
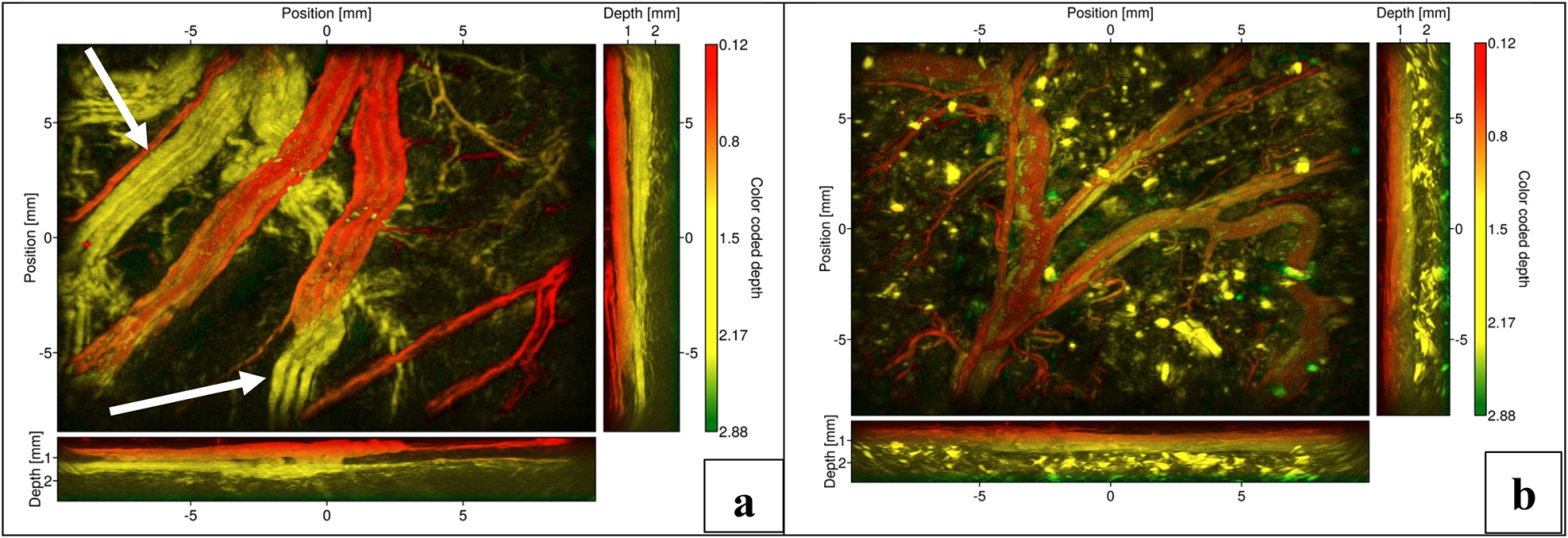
MIPs of stomach tissue (**a**) and colon tissue (**b**). Each figure presents the top-view MIPs, as well as the corresponding lateral MIPs. The arrow indicates vessel networks appearing as either triplets or pairs of parallel vessels traversing the organ (typically one artery and two veins)

Additionally, as shown in Fig. 3a, another area of the stomach demonstrated vessel networks appearing as either triplets or pairs of parallel vessels traversing the organ. These are typically composed of one artery and one or two veins. The thinness of the hollow organ walls allowed part of the incident light to diffuse through the lumen, thereby revealing the organ’s contents, as illustrated for the colon in Fig. 3b.

**Fig. 4:**
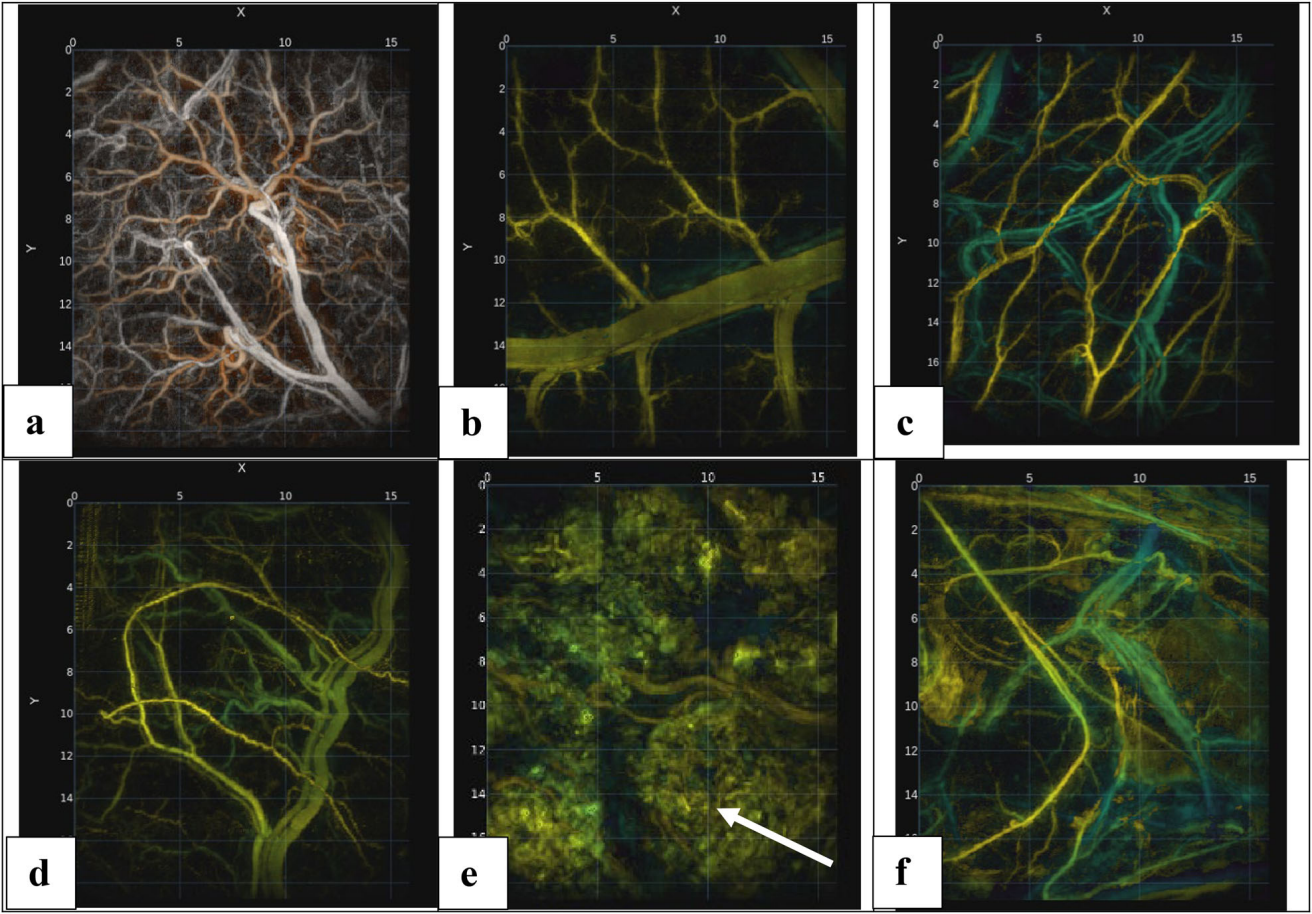
MIPs of gall bladder (**a**), small intestine (**b**), stomach (**c**), bladder (**d**), rectum (**e**), and ureter (**f**). Each figure presents the top-view MIPs. The arrow indicates the contents of the hollow organ. In the case of the rectum, it corresponds to stool, which exhibits optical absorption

Figure 4 shows the MIP of the PATI for the gall bladder, small intestine, stomach, bladder, rectum, and ureter. For all the hollow organs tested, PATI evaluation allowed for the assessment of the entire wall thickness of each organ. No hollow organ exhibited optical absorption that limited the evaluation of the full wall thickness.

### Solid organs

**Fig. 5:**
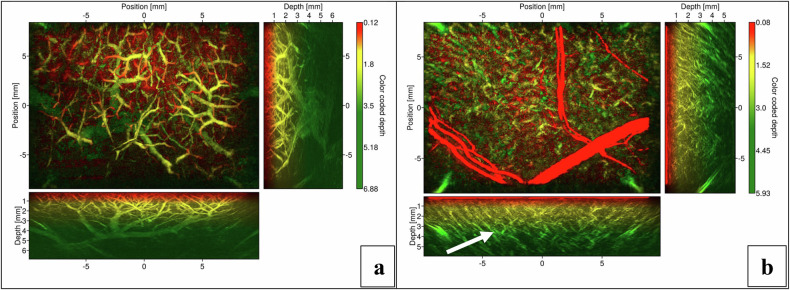
MIPs of liver tissue (**a**) and kidney tissue (**b**). Each figure presents the top-view MIP, as well as the corresponding lateral views. The arrow indicates the renal vessels in the form of linear structural features extending from the surface into deeper layers of the tissue. These vessels anatomically correspond to the interlobular arterioles (radiate part of the renal cortex)

The liver and kidney posed challenges for optical imaging modalities due to their high optical absorption, which limited light penetration and imaging depth. Despite these limitations, relatively deep penetration (approximately 6 mm) was achieved, enabling clear visualization of internal structures, as depicted in the lateral MIPs in Fig. 5a. In comparison, kidney tissue, shown in Fig. 5b, presented more linear structural features extending from the surface into deeper layers of the tissue. These images highlighted a limitation of MIP representation: it may not provide a comprehensive view of volume features when structures are distributed throughout the volume and exhibit similar amplitudes. For a better overall visualization of the full branching structure of the liver (Appendix S2) and kidney (Appendix S3), readers are referred to the 3D volume rendering video provided in the supplementary material.

**Fig. 6:**
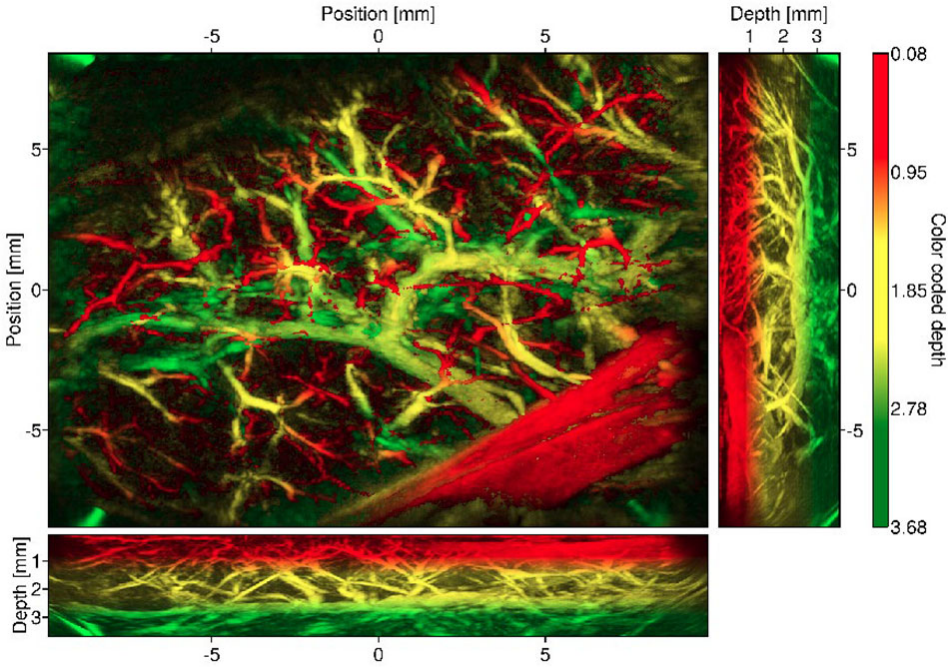
MIPs of pancreas tissue: top-view and corresponding lateral views

The pancreas exhibited a complex, heterogeneous structure composed of loosely packed lobes, contrasting with the liver’s denser and more homogeneous architecture. Pancreatic tissue demonstrated low but non-negligible optical absorption and strong scattering, rendering internal features indistinguishable to the naked eye. However, the PATI image presented in Fig. 6 successfully visualizes the intricate arborescent vascular structure supplying a large pancreatic lobe. Finally, the MIP of the PATI for the liver pedicle is presented in Appendix S4. The hepatic pedicle was a solid but thin structure with low optical absorption, allowing for the assessment of its entire thickness.

## Discussion

Our study is one of the first to provide an evaluation of intra-abdominal organs using PATI. Moreover, it is the first to achieve this with an integrated system designed for clinical use.

Compared to previous studies on *in vivo* photoacoustic imaging using FPI ultrasound sensors, the experimental conditions in this work are closer to standardized clinical operating conditions. The integration of photoacoustic imaging with an FPI ultrasound sensor into a transportable and flexible platform represents a significant advancement toward the clinical application of high-resolution photoacoustic tomography. This system enables the imaging of vascular networks and potentially any PA-responsive molecules with good resolution over a large volume (approximately 19 × 16 × 10 mm^3^). Acquisitions performed during this imaging session provided clear visualizations of tissue anatomical features. However, a comprehensive study on repeatability and reproducibility is required to rigorously evaluate the system’s reliability. Among the range of imaged organs, the reconstructed volumes of optically absorbent tissues (*e.g*., liver and kidney) are particularly noteworthy, revealing inner and deep structures that, to our knowledge, have not been observed before in PATI. Specifically, the large bandwidth of the sensing technology allows for the visualization of the multiscale complexity of the liver’s arborescent structures, from the largest vessels (approximately 1.5 mm in diameter) to the current imaging limit (< 100 µm). Thanks to this and the high image contrast, the user can directly identify the 3D volume structure without specific training to interpret the images. PATI is particularly compelling as current data suggest that this imaging modality is potentially the only one capable of providing intraoperative 3D visualization of the vascular network with depth information, using a contact probe and without the need for contrast agents. At present, no other imaging modality offers this capability. This capability of PATI could enable numerous applications in surgery and medicine. Table 1 summarizes the various potential applications of PATI.

**Table 1:** Potential indications of photoacoustic imaging in surgery and medical fields

Surgical or medical fields		Indications
Surgery	Plastic surgery	Assessment of vascularization in skin or muscle flaps.
	Transplant surgery	Evaluation of organ reperfusion after transplantation.
	Digestive and visceral surgery	Assessment of gastrointestinal tract vascularization before performing digestive anastomosis.
	Vascular surgery	Evaluation of microcirculation to determine amputation levels. Assessment of capillary density to identify collateral circulation.
Oncology	Radiotherapy	Determination of the irradiation field, integrating tumor neovascularization. Early evaluation of treatment response.
	Oncology	Early assessment of response to systemic treatments (*e.g*., melanomas). Personalized medicine based on treatment response.
	Oncologic surgery	Evaluation of tumor lesion extent to adapt surgical margins. Assessment of lymphatic drainage territory as part of the sentinel lymph node technique.
Medicine	Vascular medicine	Evaluation of capillary density in microangiopathy (*e.g*., diabetic foot).
	Dermatology	Assessment of response to photodynamic therapy.

PATI presents several challenges to be addressed in the future. PATI does not yet accurately and quantitatively differentiate between oxygen-saturated hemoglobin and deoxygenated hemoglobin, making it impossible to distinguish between arteries and veins or, conversely, ischemic tissue. This is mainly due to the complexity of establishing an accurate and robust spectral unmixing algorithm. Moreover, while the large bandwidth of the sensor grants visualization of structures of varying dimensions in a single acquisition with consistent quality, achieving the highest resolution (< 50 µm) was not always possible due to breathing-induced organ displacement exceeding one centimeter for liver and stomach. Additionally, peristaltic muscle contractions introduced blurriness in some images, such as those of the small intestine or ureter. Shorter acquisition times would mitigate these issues, reducing motion blur, minimizing operator-induced artifacts, and enabling access to rapid physiological changes. Faster acquisition would also facilitate multispectral imaging by allowing rapid sequential image superimposition. Improvements in acquisition speed could be achieved by increasing the number of simultaneous channels (32 in the current setup), the excitation laser’s repetition rate, and the wavelength switching speed. Alternatively, post-acquisition blur compensation algorithms need to be investigated. Considering that the contact of the sensor is not broken, out-of-plane movement should be minimal, and only transverse movements are expected [24].

From a practical point of view, further challenges remain to be addressed. Some organs, such as the kidneys, were challenging to image due to the probe’s size. Further miniaturization and design adaptations would enhance both imaging performance and user experience. For certain cases, future developments will focus on implementing a laparoscopic modality to support minimally invasive procedures. Additionally, laser safety glasses used during the experiments significantly distorted the perception of red wavelengths, causing blood to appear black. This absence of red coloration poses a challenge for clinicians accustomed to relying on tissue color during surgery. Several solutions can be considered. If only anatomical features are of interest, a single-wavelength laser can be enough for imaging. A single-wavelength laser—such as a 1,064-nm source—could be sufficient, offering greater imaging depth while allowing the use of high-visible-light transmission safety goggles. Alternatively, to eliminate the need for safety goggles entirely, low-power, high-pulse repetition frequency laser diodes are being explored. Finally, in its current configuration, the system is more easily operated with a clinician handling the imaging probe and positioning the subject, while an assistant controls the machine. To enable true single-operator use, acquisition triggers are being integrated directly into a new handheld probe design, along with embedded reconstruction presets for automatic, optimized imaging.

Our study has several limitations. The first is that it is an exploratory study conducted on a single animal. We do not know whether the image quality may differ from one individual to another. Experiments will need to be repeated to quantify image reproducibility. Additionally, this analysis was conducted on a porcine model, and we currently do not know whether the image quality would be the same in a human model.

The image analysis is solely qualitative. Thus, the analysis presented in this study is based only on the images interpreted according to the anatomical knowledge of the surgical team. We were unable to perform histological analysis of the evaluated area to confirm the correlation between PATI and reference histological analysis. The histological analysis could not be performed due to the risk of contaminating the surgical field (ancillary study on transplantation). Furthermore, there is no internal indicator on the platform that can attest to the reliability of PATI and its reproducibility through a deformation index. Although these limitations are present, PATI evaluations appear to be consistent with the known anatomical and histological representations of abdominal viscera.

Finally, one of the challenges of PATI is the issue of organ exposure thresholds to laser radiation. To minimize the risk of burns, we adhered to the skin laser exposure thresholds. No macroscopic burns were observed during the experiment. However, we also lack histological analysis to confirm the integrity of tissues after PATI. Future research should focus on conducting safety studies and defining maximum radiation thresholds for internal organs.

PATI is a highly promising modality for clinical applications. However, its widespread adoption is hindered by perceived complexity and the limited availability of convenient, portable, and reliable systems. In this study, we present *in vivo* imaging of porcine abdominal organs using a fully integrated photoacoustic system that utilizes FPI ultrasound detection. Our results demonstrate consistent 3D image quality across most abdominal organs, even when operated by an inexperienced user. This highlights the system’s strong potential for clinical guidance and vascularization assessment of organs. We successfully showcase the system’s ability to image blood vessels with diameters ranging from several millimeters down to less than 100 µm. The system provides valuable guidance for surgeons, as each organ exhibits a distinct vascular network signature. To further advance the technology, acquisition speed improvements will be essential to enable spectral unmixing and approach real-time imaging capabilities. Additionally, the development of a rigid laparoscopic version would unlock further clinical applications and support minimally invasive procedures.

## Supplementary information

**Additional file 1: Appendix S1** Spleen acts as a blood filter and reservoir, and consequently presents high optical absorption, limiting IR light penetration in the tissue. Strong arguments tend to indicate the presence of an open microcirculation and thus a possible diffuse appearance of the smaller features. On the reconstructed photo images, distinguishable features could only be identified down to 3 mm. This figure presents maximum intensity projections of thin slices (four samples/0.15 mm thick) of the superior layers of spleen tissue. The topmost layer presents a porous-like structure whose cavities are filled by granular features, as visible in the second image. The intensity variations present as three large vertical bands introduced in the “Materials and methods” are clearly visible. **Appendix S2** 3D volume rendering visualization of the full branching structure of the liver. **Appendix S3** 3D volume rendering visualization of the full branching structure of the kidney. **Appendix S4** Maximum intensity projections of liver pedicle tissue. Each figure presents the top-view maximum intensity projection, as well as the corresponding lateral views.

Appendix S2.

Appendix S3.

## Data Availability

The data that support the findings of this study are available from the corresponding author, BM, upon reasonable request.

## Abbreviations

3D: Three-dimensional
FPI: Fabry–Pérot interferometer
MIP: Maximum intensity projection
PATI: Photoacoustic tomography imaging
PRF: Pulse repetition frequency
