# Supplementary material for: In vivo photoacoustic tomography of porcine abdominal organs using Fabry–Pérot sensing integrated platform
Source: Eur Radiol Exp. 2025 Jul 9;9:65. doi: 10.1186/s41747-025-00601-1 (PMC12241532; doi:10.1186/s41747-025-00601-1)
Supplement: Supplementary file 1 — Additional file 1: Appendix S1 Spleen acts as a blood filter and reservoir, and consequently presents high optical absorption, limiting IR light penetration in the tissue. Strong arguments tend to indicate the presence of an open microcirculation and thus a possible diffuse appearance of the smaller features. On the reconstructed photo images, distinguishable features could only be identified down to 3 mm. This figure presents maximum intensity projections of thin slices (four samples/0.15 mm thick) of the superior layers of spleen tissue. The topmost layer presents a porous-like structure whose cavities are filled by granular features, as visible in the second image. The intensity variations present as three large vertical bands introduced in the “Materials and methods” are clearly visible. Appendix S2 3D volume rendering visualization of the full branching structure of the liver. Appendix S3 3D volume rendering visualization of the full branching structure of the kidney. Appendix S4 Maximum intensity projections of liver pedicle tissue. Each figure presents the top-view maximum intensity projection, as well as the corresponding lateral views. [file 41747_2025_601_MOESM1_ESM.pdf]

In vivo photoacoustic tomography of porcine abdominal organs using Fabry-Pérot sensing integrated platform

ELECTRONIC SUPPLEMENTARY MATERIAL

Appendix S1

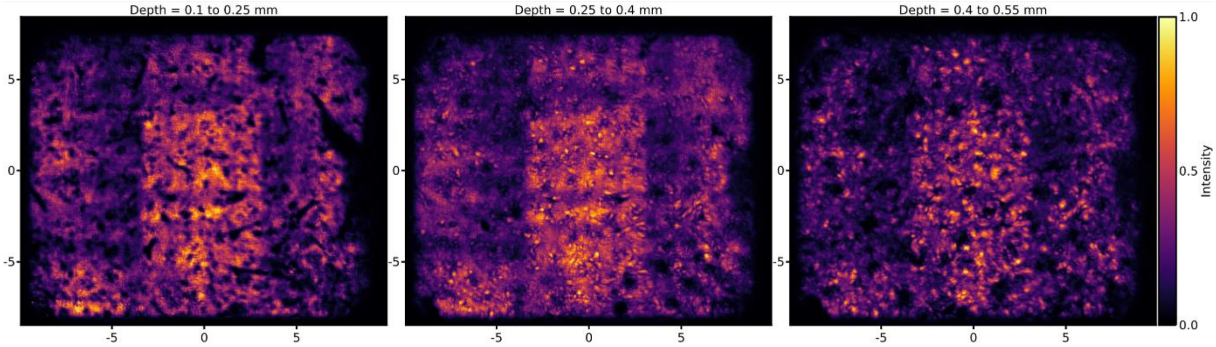

Spleen acts as a blood filter and reservoir and consequently presents high optical absorption limiting IR light penetration in the tissue. Strong arguments tend to indicate the presence of an open microcirculation and thus a possible diffuse appearance of the smaller features. On the reconstructed photo images, distinguishable features could only be identified down to 3mm depth. This figure presents maximum intensity projections of thin slices (4 samples/ 0.15mm thick) of the superior layers of spleen tissue. The topmost layer presents a porous-like structure which cavities are filled by granular features as visible in the second image. The intensity variations present as three large vertical bands introduced in Materials and method are clearly visible.

Appendix S2

3D volume rendering visualization of the full branching structure of the liver.

Appendix S3

3D volume rendering visualization of the full branching structure of the kidney.

Appendix S4

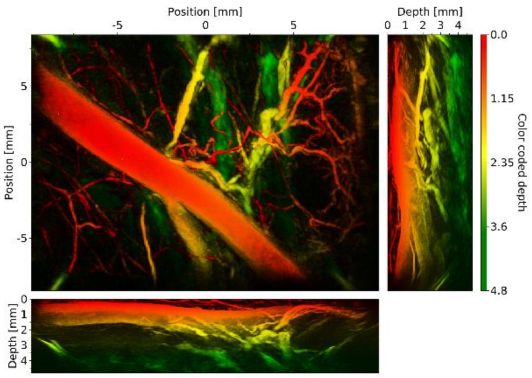

Maximum intensity projections of liver pedicle tissue. Each figure presents the top-view maximum intensity projection as well each corresponding lateral views.
